# Differential effects of Smad3 targeting in a murine model of chronic kidney disease

**DOI:** 10.1002/phy2.181

**Published:** 2013-12-15

**Authors:** Terese Kellenberger, Søren Krag, Carl Christian Danielsen, Xiao‐Fan Wang, Jens Randel Nyengaard, Lea Pedersen, Chuanxu Yang, Shan Gao, Lise Wogensen

**Affiliations:** 1 Research Laboratory for Biochemical Pathology, Aarhus University Hospital, Institute of Clinical Medicine, University of Aarhus, Aarhus, Denmark; 2 Department of Connective Tissue Biology, Institute of Biomedicine, University of Aarhus, Aarhus, Denmark; 3 Pharmacology and Cancer Biology, Duke University Medical Center, Durham, NC; 4 Stereology and Electron Microscopy Laboratory, Centre for Stochastic Geometry and Advanced Bioimaging, Institute of Clinical Medicine, University of Aarhus, Aarhus, Denmark; 5 Department of Molecular Biology, University of Aarhus, Aarhus, Denmark

**Keywords:** Extracellular matrix, fibrosis, matrix metalloproteinase, transforming growth factor‐*β*1

## Abstract

Transforming growth factor (TGF)‐*β*1 has a pivotal role in the pathogenesis of progressive kidney diseases that are characterized by fibrosis. The main intracellular signaling pathway of TGF‐*β*1 is the Smad system, where Smad2 and Smad3 play a central role in transcriptional regulation of target genes involved in extracellular matrix (ECM) metabolism. This study analyzes the hypothesis that blockade of Smad3 attenuates the development of TGF‐*β*1‐driven renal fibrosis. This was examined in vivo in a transgenic model of TGF‐*β*1‐induced chronic kidney disease with Smad3 or without Smad3 expression and in vitro in mesangial cells and glomerular endothelial cells with Smad2/3 inhibitors or Smad3‐knockdown. Electron microscopy was used for evaluation of morphological changes, real‐time polymerase chain reaction for detection of RNA expression, and immunohistochemistry for localization of ECM components. Matrix metalloproteinase (MMP) level was assessed by gelatin zymography electrophoresis and located by in situ zymography. The results show TGF‐*β*1‐induced mesangial matrix expansion, tubulointerstitial fibrosis, and tubular basement membrane thickening that are attenuated by Smad3 deletion, whereas TGF‐*β*1‐induced glomerular basement membrane thickening is not shown. The amount and distribution profile of MMP‐2 may suggest a role of the enzyme herein. We conclude that Smad3 targeting is not exclusively beneficial as Smad3 has diverse transcriptional regulatory effects in different cell types in the kidney.

## Abstract (precis)

Deletion of Smad3 protects the kidney from developing transforming growth factor (TGF)‐*β*1‐induced tubulointerstitial fibrosis, mesangial matrix expansion, and tubular basement membrane thickening, but not glomerular basement membrane thickening. The favorable effects of Smad3 deficiency can be explained by reduced deposition of collagen subtypes. The cell‐specific changes of matrix metalloproteinase expression can be a result of altered TGF‐*β*1 signaling.

## Introduction

Transforming growth factor (TGF)‐*β*1 is a key element in the pathogenesis of renal fibrosis. It is elevated in various kidney diseases including diabetic nephropathy, the single most common cause of renal replacement therapy worldwide (Ashcroft et al. 1999; Locatelli et al. 2004; Marshall 2004; Yamamoto et al. 1993). TGF‐*β*1 acts through a diversity of pathways (Derynck and Zhang 2003), which reflects its multifunctional involvement in processes such as the regulation of extracellular matrix (ECM) production and degradation, induction of fibroblast to myofibroblast differentiation, and epithelial‐to‐mesenchymal transition (Wynn 2008). The main intracellular mediator of TGF‐*β*1 signaling is the Smad system (Goumans et al. 2009; Massague 1996). By activating the TGF‐*β*1 transmembrane serine/threonine kinase receptor II and I, cytoplasmatic Smad2 and Smad3 become phosphorylated and translocate to the nucleus to activate the transcription of target genes in a cell‐type‐specific manner (Derynck et al. 1998). Smad2 and Smad3 are >90% homologous in their structure (Yagi et al. 1993), but their functions differ especially in embryonic development and in the context of tissue repair and fibrosis (Meng et al. 2010). This may be explained by the absence of sequence‐specific DNA‐binding activity in the MH1 domain in Smad2 (Roberts et al. 2003). It is postulated that Smad2 and Smad3 can substitute each other if one of the genes is deleted or that a third pathway will take charge. This may have unexpected biological consequences in vivo (Goumans et al. 2003). Several studies have investigated the role of Smad2 and Smad3 in the pathogenesis of different renal diseases in vivo (Fujimoto et al. 2003; Inazaki et al. 2004; Meng et al. 2010; Nath et al. 2011; Sato et al. 2003; Wang et al. 2007; Warner et al. 2012; Zhou et al. 2010). These studies reveal that deletion of the Smad3 gene protects against renal tubulointerstitial fibrosis (TIF) and mesangial matrix expansion, but the impact of Smad3 on other characteristics of renal fibrosis such as glomerular basement membrane (GBM) thickening, atrophy, and albuminuria cannot be uniformly confirmed. Common for these in vivo studies is that the role of Smad3 on the matrix metalloproteinase (MMP)/tissue inhibitors of metalloproteinase (TIMP) system is unexplored.

Until recently, it was traditionally believed that MMPs were purely antifibrotic due to ECM degrading capabilities. Thus, high levels of MMPs were regarded as beneficial in the healing process. New investigations reveal, however, that MMPs are important players in inflammation, cell proliferation and death, and epithelial‐mesenchymal transition as well (Tojo et al. 2005). Therefore, the effect of MMPs on the balance between ECM deposition and ECM degradation in vivo is multifaceted and may be difficult to predict. This is substantiated by the finding that low levels of MMP expression or MMP inhibition is beneficial in being associated with improved kidney morphology rather than ECM deposition (Tan and Liu 2012; Wang et al. 2010; Williams et al. 2011). These observations underline the importance of clarifying the regulations of MMPs in fibrotic kidney diseases.

Thus, the role of Smad3 on MMP regulation, GBM, and tubular basement membrane (TBM) remodeling in chronic kidney disease in vivo needs to be clarified. To address this, we have employed transgenic mice, with slowly progressing TGF‐*β*1‐induced kidney disease, with and without an intact Smad3 gene.

## Material and Methods

### Generation of Smad3 KO mice

Smad3 heterozygotic (Smad3 HT) animals (Datto et al. 1999) were backcrossed for more than seven generations with transgenic mice (BALB/cA) overexpressing porcine TGF‐*β*1 (pTGF‐*β*1) under control of the Renin‐1^c^ promoter (Wogensen et al. 1999). Smad3 knockout (Smad3 KO) mice, Smad3 HT and Smad3 wild‐type (Smad3 WT) mice were obtained in the expected 25%:50%:25% Mendelian ratio. Smad3 WT and Smad3 KO nontransgenic (non‐TGF‐*β*1) and transgenic (TGF‐*β*1) mice were used in the experiments. The genotype of the mice was established by polymerase chain reaction (PCR) on DNA from tail biopsies with primers targeting the terminator sequence of the transgene (Wogensen et al. 1999). The mice were housed at the animal facility at the University of Aarhus and handled according to the guidelines and procedures approved by the Animal Experiments Inspectorate, Denmark (2005/562‐8 and 2012‐15‐2935‐00003) and the Danish Working Environment Service (20010011479/12). The animals were kept at 21°C on a 12‐h day/night cycle and were given free access to standard chow and water. Unless otherwise stated, 8‐week‐old sex‐matched animals were killed by cervical dislocation and used for all analyses. Eight‐week‐old mice were chosen as the morphological changes exhibit a gradual progression with age and the changes in the glomeruli may be too abundant to be evaluated properly by electron microscopy (Wogensen et al. 1999).

### Collection of material

Spot urine was collected from the mice before sacrifice. One kidney was sampled and immersion fixed for stereology in Tyrode's buffer containing 1% glutaraldehyde and 3% paraformaldehyde. The other kidney was divided into three parts: one was placed in OCT‐compound (Sakura Finetek, Tokyo, Japan) and frozen in liquid nitrogen; one was fixed in paraformaldehyde and embedded in paraffin; and one was frozen in liquid nitrogen and stored at −80°C.

### Assessment of the urinary albumin/creatinine ratio

The urinary albumin concentration was measured using a commercial ELISA specific for mouse albumin diluted 1:500 to 1:5000 (Bethyl Laboratories Inc, Montgomery, TX). Creatinine was measured on a colorimetric assay based on Jaffé reaction (undiluted and 1:10) (Cayman Chemical, Ann Arbor, MI).

### Glomerular volume

The kidney was cut into 1‐mm slices, and every third slice was chosen by systematic, uniformly random sampling and embedded in the same glycol methacrylate plastic block. A 15‐*μ*m dissector pair and one 3‐*μ*m section were cut through the center of the block and stained with periodic acid‐Schiff. The counting was done using a microscope and CAST software (Olympus, Ballerup, Denmark).

The glomerular density (*N*_*v*_(glom/kidney)) in a dissector was estimated as:
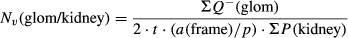


In this formula, *Q*^*−*^ is the number of glomeruli counted, *t* is thickness of the dissector sections, *a*(frame)/*p* is the area associated per test point, and *P*(kidney) is the number of points overlaying the kidney. The step length is 1400 and 1275 *μ*m. Total magnification on the screen is 152 and *a*(frame)/*p *= 82,700 *μ*m^2^.

The volume fraction of glomeruli (*V*_*v*_(glom/kidney)) was estimated on the 3‐*μ*m sections using point counting with two points for the total kidney and 60 points for the glomeruli. The mean glomerular volume (*V*_*n*_(glom)) was then calculated as:
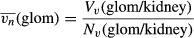


### Mesangial volume and GBM thickness

From the remaining slices, small kidney biopsies were punched randomly from the cortex using a plastic grid with equidistantly spaced holes. These were embedded in Epon, contrasted using an automated stainer (EMstain; Leica, Wetzlar, Germany) and 2–3‐*μ*m sections were cut until at least one glomerulus appeared. Using an ultramicrotome (Leica) 50‐nm sections were cut. Using a Phillips CM10 electron microscope with a Kodak 1.6 camera (Kodak, Rochester, NY) and analysis software version 3.1 (Soft Imaging Systems, Münster, Germany), the glomeruli were photographed. The mesangial volume fraction was point counted on a 3400× original magnification using a point grid (1:9). The glomerular area was defined as the polygon obtained when the outer capillary profiles were connected by straight lines. At 7900× original magnification, the GBM thickness was measured where it intersected with test lines, and the harmonic mean was calculated (Gundersen et al. 1978).

### Proximal TBM thickness

Some of the above‐mentioned kidney biopsies were embedded in 5% agar in an isector to generate isotropic sections (Nyengaard et al. 1997). They were embedded in Epon, ultrathin sections were cut as mentioned above and profiles of proximal tubules were identified using a FEI Morgagni electron microscope with a SIS 3 digital camera (Olympus Soft Imaging Solution GmbH, Münster, Germany). The proximal TBM thickness was measured on the isotropic sections at 8900× original magnification and the harmonic mean was calculated.

### Total collagen content

The renal content of collagen was estimated by determination of the quantity of hydroxyproline as described (Danielsen and Andreassen 1988). In short, kidney slices were defatted in chloroform: methanol 2:1, including protease inhibitors, for 21 h at 4°C. Then 200 *μ*L 6 mol/L HCl was added to 10 mg of dried and pulverized tissue, and hydrolyzed at 118°C for 18 h. This was neutralized using 120 *μ*L 10 mol/L NaOH. After centrifugation, the content of hydroxyproline in the supernatant (1:75) was determined in the presence of chloramine T, perchloric acid, and Ehrlich's reagent. The standard curve ranged from 0.2 to 16 *μ*g/mL. The collagen content was calculated from total hydroxyproline using 7.46 as correction factor (Neuman and Logan 1950).

### mRNA expression by real‐time RT‐PCR

Total RNA was extracted from mice kidneys using Trizol reagent (Invitrogen, Carlsbad, CA). For each sample, 1 *μ*g RNA was reverse‐transcribed using RT‐Superscript III (Invitrogen) and PCR was carried out by using iCycler™ (Bio‐Rad, Hercules, CA). All the reactions were performed with SYBR green (Bio‐Rad) under the same conditions as follows: 95°C for 5 min followed by 45 cycles 30 sec at 95°C (denaturation), 1 min at 60°C (annealing), 45 sec at 72°C (extension), and 5 min at 72°C (final extension). A standard curve (1:5, 1:25, 1:125, and 1:625) was mixed from all the cDNA samples and included in each PCR reaction. To quantify the expression of the genes, they were compared to the housekeeping gene GAPDH (DNA Technology, Risskov, Denmark). Before running the experiments, the amplification products were controlled by melting point determination.

The primer pairs used in the study were: collagen *α*1(III): forward 5′‐TGGTTTCTTCTCACCCTTCTTC‐3′, reverse 5′‐TGCATCCCAATTCATCTACGT‐3′; collagen *α*1/*α*2(IV): forward 5′‐CACCATAGAGAGAAGCGAGATGTTC‐3′, reverse 5′‐GGCTGACGTGTGTTCGC‐3′; collagen *α*3(IV): forward 5′‐ACGGTGTGTTCCTTGTCTCC ‐3′, reverse 5′‐TTCTCTTCACGGTGTGCTTG‐3′; GAPDH: forward 5′‐ATGTTCCAGTATGACTCCACTCACG‐3′, reverse 5′‐GAAGACACCAGTAGACTCCACGACA‐3′; megalin: forward 5′‐CAGTGGATTGGGTAGCAGGA‐3′, reverse 5′‐GCTTGGGGTCAACAACGATA‐3′; MMP‐2: forward 5′‐CCCCATGAAGCCTTGTTTACC‐3′, reverse 5′‐TTGTAGGAGGTGCCCTGGAA‐3′; MMP‐9: forward 5′‐AGACCAAGGGTACAGCCTGTTC‐3′, reverse 5′‐GCCTACACCCCAGTCATGGA‐3′; TGF‐*β*1 (porcine): forward 5′‐GCCTGCTGAGGCTCAAGTTA‐3′, reverse 5′‐ATCAAAGGACAGCCACTCCG‐3′; TGF‐*β*1 (murine): forward 5′‐ACCTTGGTAACCGGCTGC‐3′, reverse 5′‐TCCTTGGTTCAGCCACTGC‐3′; TIMP‐1: forward 5′‐GGCCCGTGATGAGAAACTCTT‐3′, reverse 5′‐GCCTACACCCCAGTCATGGA‐3′; TIMP‐2: forward 5′‐AGGAGATGTAGCAAGGGATCA‐3′, reverse 5′‐GAGCCTGAACCACAGGTACCA‐3′.

### Western blot analysis

Proteins from renal tissue (4‐month‐old mice) were extracted with lysis buffer and analyzed by Western blotting. Twenty micrograms of denatured proteins were loaded in equalized amounts, electrophoresed under reducing conditions on a 4–12% criterion XT Bis‐Tris gel (Bio‐Rad Laboratories) and transferred onto a polyvinylidene difluoride membrane for the detection of Smad3. The membranes were blocked in 2% bovine serum albumin (BSA) dissolved in Tris‐buffered saline, and incubated overnight with Smad3 rabbit monoclonal antibody (1:1000, catalog no. 9523; Cell Signaling Technology, Danvers, MA). The western signal was developed with horseradish peroxidase‐tagged secondary antibody and luminal‐based enhanced chemiluminescent substrate (Thermo Scientific, Rockford, IL). Monoclonal *α*‐tubulin antibody (1:500, catalog no. CP06; Calbiochem, Darmstadt, Germany) was used for detection of housekeeping protein.

### Gelatin zymography

Gelatin zymography was used to measure the MMP‐2. The wet weight of the kidney tissue was recorded. The samples were homogenized in 50 mmol/L Tris/HCl (pH 7.4) with 10 mmol/L CaCl_2_, 0.05% Brij 35, and 1 mmol/L phenylmethylsulfonyl fluoride (PMSF) overnight at 4°C. Following centrifugation, the pellet was re‐extracted overnight at 4°C, followed by three heat extractions (60°C for 4 min). The extracts from each animal (in total 40 *μ*L/mg wet weight) were pooled and subjected to gelatin zymography as described (Danielsen et al. 1998). In short, extracts were diluted twofold with sample buffer (4% sodium dodecyl sulfate, 20% glycerol, 0.1% bromophenol blue, 62.5 mmol/L Tris/HCl, pH 6.8), incubated at 37°C for 15 min before loading onto the gels (0.06 mg tissue per lane). Each gel was loaded with extracts from mice from each group together with MMP‐2 (Biogenesis, Poole, U.K.) and MMP‐9 (Kjeldsen et al. 1993) standards. Following electrophoresis, the gels were washed in 2.5% Triton X‐100 for 2 x 30 min. After a brief wash in 1 *μ*mol/L ZnCl_2_, 5 mmol/L CaCl_2_, and in 10 mmol/L Tris/HCl, pH 7.5, the gels were incubated in the same buffer plus 25 *μ*mol/L PMSF for 20 h at 37°C during gentle agitation. The gels were stained in a 1:1 mixture of staining (0.5 g Coomassie Brilliant Blue, 250 mL methanol, 100 mL acetic acid, 150 mL dH_2_O) and destaining (300 mL methanol, 10 mL formic acid, and 700 mL dH_2_O) solution for 1 h, followed by incubation in destaining solution alone for 1 h. The MMP‐2 was measured by analyzing the lysis band intensity using the UVP BioSpectrum^®^500 Imaging System (Ultra‐Violet Products Ltd, Cambridge, U.K.). Each extract was repeatedly run on three to four gels.

It should be underlined that the MMP‐2 level in 4‐month‐old Smad3 WT mice was estimated on tissue from our ordinary breed of TGF‐*β*1 transgenic BALB/cA mice with intact Smad3. Thus, the animals are not generated from the HT breeding. However, there are no obvious differences in several tested parameters, including the mRNA expression of MMP‐2, when comparing ordinary WT mice versus Smad3 WT mice from the HT breeding: MMP‐2 (ratio): 0.74 ± 0.37 (*n *= 16/group) versus 0.77 ± 0.44 (*n* = 10/group) in non‐TGF‐*β*1 animals and 1.99 ± 1.01 (*n* = 14/group) versus 2.32 ± 1.24 (*n* = 10/group) in TGF‐*β*1 mice.

### In situ zymography

FITC‐labeled DQ‐gelatin (EnzCheck; Molecular Probes, Eugene, OR) was used as substrate. One percent low melting point agarose melted in buffer (50 mmol/L Tris HCl, 150 mmol/L NaCl, 5 mmol/L CaCl, and 0.05% Brij35) was mixed (1:4) with DQ‐gelatin substrate (1 mg/mL). The warm solution was spread on preheated glass slides (37°C). The mixture was allowed to gel at room temperature in the dark. A fresh cryostat section (6 *μ*m) was placed on top of the homogeneous gel, and 50 *μ*L Tris‐buffer with PMSF (1 mmol/L) was added and overlaid with a coverslip. The sections were placed in a dark humid chamber at 37°C for 1–2 h. Sections were examined under a fluorescence microscope. MMP activity was observed as a green fluorescence in the sections and score after the amount of staining: minor (Akis and Madaio 2004), moderate (Ashcroft et al. 1999), and high appearance (Danielsen and Andreassen 1988). Addition of MMP inhibitor 1.10‐phenanthroline or a mixture of inhibitors of metallo, cysteine, aspartate, and serine proteases decreased the gelatinase activity.

### Histology and immunohistochemical staining

Paraffin sections were stained with Mallory's trichrome to evaluate the deposition of collagen fibers. Trichrome sections were semiquantified by two blinded observers using the following scoring for the staining: minor appearance (Akis and Madaio 2004), moderate appearance (Ashcroft et al. 1999), and high appearance (Danielsen and Andreassen 1988) (whole sections were observed).

Localization of collagen *α*1(III) was performed in 4‐*μ*m paraffin sections using microwave‐based antigen retrieval technique (10 min in citrate buffer, pH 6.0) with a polyclonal anti‐rabbit collagen *α*1(III) antibody (1:100; GenWay Biotech, Inc., San Diego, CA). Localization of collagen *α*1/*α*2(IV) was performed on cryosections with a polyclonal anti‐goat collagen *α*1(IV) antibody (1:30; Southern Biotechnology, Birmingham, AL). The incubation with the primary antibody was followed by reaction with biotinylated secondary antibodies (1:200; Vector Laboratories, Burlingame, CA), which then was detected using the ABC kit (catalog no. PK‐4000; Vector Laboratories) and 3,3′‐diaminobenzidine (Sigma‐Aldrich, St. Louis, MI). Omission of the primary antibodies served as negative controls.

### Immunofluorescence

Acetone‐fixed, frozen sections were unmasked (0.1 mol/L glycine pH 2.2/6 mol/L urea) and blocked with 3% BSA/3% normal goat serum followed by incubation with rat monoclonal antihuman collagen *α*4(IV) (1:100, catalog no. 7074; Chondrex, Redmond, WA). The sections were incubated with rhodamine‐conjugated anti‐rat IgG (1:200; Santa Cruz Biotechnology, Dallas, TX) and mounted with Vectashield mounting medium (Vector Laboratories).

### Mesangial cell and endothelial cell cultures

A mouse immortalized mesangial cell line (SV40 MES 13) was obtained from American Type culture collection (LGC Promochem, Boras, Sweden). The cells were maintained in a 3:1 mixture of Dulbecco's modified Eagle's medium (DMEM) and Ham′s F12, 5.5 mmol/L glucose, 5% fetal bovine serum (FBS). A murine immortalized glomerular endothelial cell line, kindly provided by Akis and Madaio (2004), was maintained in 75 cm^2^ gelatin‐coated (0.5%) bottles in DMEM containing 10% (v/v) human serum, 2 mmol/L l‐glutamine, 25 *μ*g/mL endothelial cell growth supplement (Sigma‐Aldrich), and 0.1 mg/mL heparin sodium salt, at 37°C in a humidified atmosphere of 5% CO_2_. The phenotype of the endothelial cells was confirmed by the appearance of a cobblestone‐like monolayer as evaluated by phase‐contrast microscopy and detection of von Willebrand factor and CD34. For the experiments, 160,000 mesangial cells and 150,000 endothelial cells were seeded in 12‐well plates. Recombinant human TGF‐*β*1 (2 ng/mL) (R&D systems, Minneapolis, MN) was added alone or in combination with the Smad2/3 inhibitor A83‐01 (1 *μ*mol/L). Following an 18‐h incubation period, RNA was extracted using Trizol (Invitrogen). Each experiment was repeated at least two times.

### Validation of murine Smad3 small interfering RNA

Mouse embryonic fibroblast (NIH 3T3) and macrophage (J774.A1) cell lines were used for validation of small interfering RNA (siRNA) efficacy. Genepharm (Shanghai, China) provided three siRNAs against murine Smad3 and one negative control siRNA. The sequences were Smad3‐murine‐956 (siSmad3‐1): 5′‐CAGCACACAAUAACUUGGATT‐3′; Smad3‐murine‐4492 (siSmad3‐2): 5′‐CUGCCGCUUUCGUAUUUAUTT‐3′; Smad3‐murine‐4795 (siSmad3‐3): 5′‐GGGAACUUCAAAUGGAAAUTT‐3′; negative control siRNA (siNC): 5′‐UUCUCCGAACGUGUCACGUTT‐3′. siRNA against enhanced green fluorescent protein (EGFP) (siEGFP): 5′‐GACGUAAACGGCCACAAGUUC‐3′ (Ribotask, Odense, Denmark). According to standard protocols, all siRNAs were transfected via Lipofectamine™2000 (Invitrogen) for NIH 3T3 cells and *Trans*IT‐TKO (Mirus Bio., Madison, WI) for J774.A1 cells at a final concentration of 50 nmol/L siRNA (6‐well plates). After overnight incubation, the media were replaced with fresh cell culturing media. The cells were harvested at 48 h post transfection. Quantitative real‐time RT‐PCR was performed for evaluation of Smad3 mRNA level using a Mx4000^®^ Multiplex Quantitative PCR System (Stratagene, Copenhagen, Denmark). The Smad3 mRNA expression level was normalized against the level of *β*‐actin.

### Transfection of mesangial cells and endothelial cells with siRNA

Immortalized mesangial cells (SV40 MES 13) and immortalized glomerular endothelial cells were transfected using Lipofectamine™2000 (Invitrogen) according to the manufacturer′s protocol. In brief, 75,000 mesangial cells (12‐well plates) or 180,000 endothelial cells (6‐well plates) were seeded the day before transfection. The transfection mixture was prepared as follows: 6 *μ*L Lipofectamine™2000 reagent (Invitrogen) was added to 250 *μ*L DMEM without serum or antibiotics. siSmad3 or siEGFP (final siRNA concentration of 50 nmol/L) was also added to 250 *μ*L medium without serum and antibiotics. This was mixed with the Lipofectamine™2000 solution for 15 min at RT before adding to the cells. After 4 h, the cells were supplemented with 5% FBS. The transfection medium was replaced after 20 h and the cells were maintained for further 20 h under normal culture conditions. The cells were then stimulated with recombinant human TGF‐*β*1 (2 ng/mL) (R&D systems) for 22 h. The cells were harvested and RNA was isolated using Trizol (Invitrogen). Data were obtained from three independent experiments. Results from one experiment are shown.

### Statistical analysis

All data are presented as mean ± standard deviation (SD). When more than two groups were compared, ANOVA was applied utilizing the Holm–Sidak test for adjustment of all pairwise multiple comparisons or comparisons versus a control group. In the presence of unequal variance or if the data did not follow a normal distribution, a one‐way ANOVA on Ranks was used, followed by Dunn's or Newman–Keuls test. In Dunn's ANOVA test, the relevant groups to be compared were isolated. Pairwise comparisons were evaluated using the Student's *t*‐test. Values were considered significant at *P *< 0.05. If not otherwise stated *n* = number of mice investigated/group.

## Results

### Verification of the model

**Figure 1.**
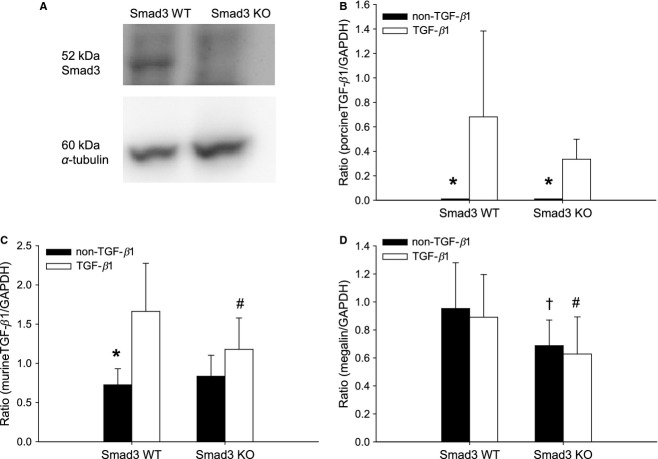
Verification of the model. (A) Western blot of total Smad3 protein in the kidneys. Total Smad3 protein was present in Smad3 WT non‐TGF‐*β*1 mice, but absent in Smad3 KO non‐TGF‐*β*1 mice. *α*‐tubulin is used as reference protein. (B) Porcine TGF‐*β*1 mRNA expression is high in the TGF‐*β*1 mice, and absent in the non‐TGF‐*β*1 mice (*P < 0.05 vs. TGF‐*β*1 mice). Porcine TGF‐*β*1 mRNA expression is similar in Smad3 WT TGF‐*β*1 versus Smad3 KO TGF‐*β*1 (Smad3 WT TGF‐*β*1 range: 0.138–1.903; Smad3 KO TGF‐*β*1 range: 0.170–0.645 (*P *> 0.05) (*n *= 5–6/group). (C) The expression of murine TGF‐*β*1 is increased in Smad3 WT TGF‐*β*1 mice (*P < 0.05), but not in Smad3 KO TGF‐*β*1 mice. Thus, reduced level of endogenous TGF‐*β*1 is seen in Smad3 KO TGF‐*β*1 versus Smad3 WT TGF‐*β*1 mice (^#^*P* < 0.05) (*n* = 6–7/group). (D) Megalin mRNA expression is reduced in Smad3 KO mice versus Smad3 WT animals (^†#^*P* < 0.05) (*n* = 14–21/group).

Smad3 was present in Smad3 WT mice, but absent in the Smad3 KO mice (Fig. 1A). As expected, there was no detectable pTGF‐*β*1 mRNA expression in non‐transgenic mice, whereas similar levels were observed in Smad3 WT and Smad3 KO TGF‐*β*1 transgenic animals (Fig. 1B). Endogenous (murine) TGF‐*β*1 mRNA expression was upregulated in Smad3 WT TGF‐*β*1 mice compared to Smad3 WT non‐TGF‐*β*1 mice (*P < 0.05) (Fig. 1C). This increase in endogenous TGF‐*β*1 expression was absent in Smad3 KO TGF‐*β*1 mice.

### Baseline characteristics of Smad3 WT and Smad3 KO mice

At 2 months of age, the body weights (BW) of Smad3 KO mice were less than that of Smad3 WT animals (*^†^*P* < 0.05) (Table 1). Overexpression of pTGF‐*β*1 reduced the BW of Smad3 WT mice (*P < 0.05), but not that of Smad3 KO mice (Table 1). The kidney weight (KW)/BW ratio was the same in all groups (Table 1). The glomerular volume (*V*_*n*_(glom)) was reduced in Smad3 KO non‐TGF‐*β*1 versus Smad3 WT non‐TGF‐*β*1 mice (*P < 0.05) (Table 1). pTGF‐*β*1 expression was without impact on *V*_*n*_(glom).

**Table 1.** Basic characteristics of WT and Smad3 KO mice with TGF‐*β*1‐induced kidney disease.

	*n*	Smad3 WT		Smad3 KO	
		non‐TGF‐*β*1	TGF‐*β*1	non‐TGF‐*β*1	TGF‐*β*1
BW (g)	10	22.8 ± 2.9	19.2 ± 2.4*	13.1 ± 4.1*	13.1 ± 2.9†
KW/BW (mg/g)	10	8.4 ± 1.1	7.6 ± 1.3	8.3 ± 0.9	7.2 ± 0.2
*V*_*n*_ (glom)(10^3^ *μ*m^3^)	7–8	101 (93–114)	113 (94–120)	58 (44–75)*	74 (62–88)
Albumin/creatinine ratio (ng/*μ*g)	9–11	44 (33–57)	25 (19–40)	89 (61–131)*	56 (37–134)†
*V*_*v*_ (mes/glom) (%)	7–9	25 ± 3.1	35 ± 6.4*	23 ± 4.7	26 ± 5.4
*V* (mes, glom) (10^3^ *μ*m^3^)	7–9	25 (24–29)	34 (32–48)	12 (11–17)	19 (15–22)†
GBM (nm)	7–9	144 ± 7.3	190 ± 14.0*	128 ± 8.0*	175 ± 25.0†
TBM (nm)	5–6	136 ± 10.1	155 ± 18.4*	135 ± 12.4	140 ± 10.0

Values are means ± SD or medians (25% and 75% percentiles). BW, body weight; KW, kidney weight; GBM, glomerular basement membrane; TBM, tubular basement membrane; TGF‐*β*1, transforming growth factor. *P < 0.05 versus Smad3 WT non‐TGF‐*β*1. ^†^*P* < 0.05 versus Smad3 WT TGF‐*β*1. ^‡^*P* < 0.05 versus Smad3 KO non‐TGF‐*β*1.

The albumin/creatinine ratio was increased twofold in Smad3 KO mice versus Smad3 WT mice (*^†^*P* < 0.05) (Table 1). pTGF‐*β*1 expression had no impact on albumin/creatinine ratio in either group. Due to a suspicion of gender‐related differences in megalin expression, the original group of mice was supplemented with an additional group of 8‐week‐old animals. We found a reduced megalin mRNA expression in Smad3 KO mice versus Smad3 WT (^†^^#^*P *< 0.05) (Fig. 1D). The megalin mRNA expression was similar within the two groups of Smad3 WT mice and within the two groups of Smad3 KO animals (Fig. 1D).

### Smad3‐deletion does not attenuate TGF‐*β*1‐mediated thickening of the GBM

**Figure 2.**
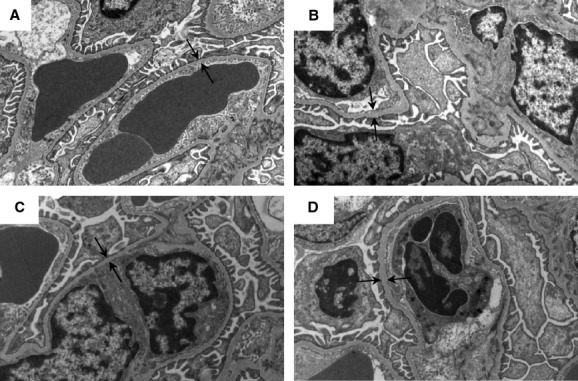
Electron microscopic photographs of the GBM from representative mice are shown: Smad3 WT non‐TGF‐*β*1 (A), Smad3 WT TGF‐*β*1 (B), Smad3 KO non‐TGF‐*β*1 (C), and Smad3 KO TGF‐*β*1 (D). Arrows indicate the GBM thickness. Final magnification: 18,400×. GBM, glomerular basement membrane.

**Figure 3.**
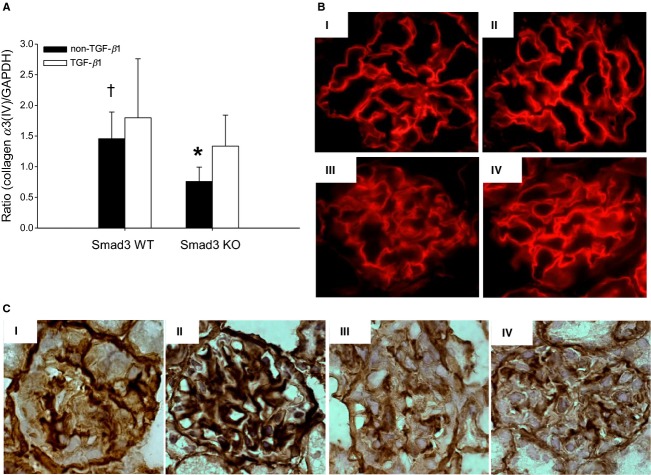
Expression and localization of collagen type IV. (A) Expression of collagen *α*3(IV) mRNA is upregulated in Smad3 KO TGF‐*β*1 mice compared with Smad3 KO non‐TGF‐*β*1 mice (*P = 0.007). A reduction in collagen *α*3(IV) mRNA is seen in the Smad3 KO non‐TGF‐*β*1 mice versus Smad3 WT non‐TGF‐*β*1 mice (^†^*P *< 0.05) (*n* = 6–7/group). (B) Immunofluorescence staining of collagen *α*4(IV) in glomerulus in Smad3 WT non‐TGF‐*β*1 (I), Smad3 WT TGF‐*β*1 (II), Smad3 KO non‐TGF‐*β*1 (III) and Smad3 KO TGF‐*β*1 (IV) mice (magnification: 100×) (*n* = 3/group). (C) Collagen *α*1/*α*2(IV) staining (brown) in glomerulus from representative mice; (I) Smad3 WT non‐TGF‐*β*1, (II) Smad3 WT TGF‐*β*1, (III) Smad3 KO non‐TGF‐*β*1, and (IV) Smad3 KO TGF‐*β*1 mice.

The thickness of the GBM is a sensitive marker of glomerulopathy (Lehmann and Schleicher 2000). As estimated using electron microscopy, the GBM was thinner in Smad3 KO non‐TGF‐*β*1 mice compared with Smad3 WT non‐TGF‐*β*1 mice (*P < 0.05) (Table 1) (Fig. 2). However, overexpression of pTGF‐*β*1 led to increased GBM thickness, irrespective of Smad3 genotype (Table 1) (Fig. 2). The enlarged GBM thickness was paralleled by biochemical changes. One of the important elements in the mature GBM is the network of collagen *α*3/*α*4/*α*5(IV) (Miner 1999; Zeisberg et al. 2002). As measured by real‐time PCR on total kidney tissue, pTGF‐*β*1 overexpression increased the level of collagen *α*3(IV) RNA in the Smad3 KO mice (*P = 0.007) (Fig. 3A). Furthermore, a significant reduction in collagen *α*3(IV) mRNA was seen in the Smad3 KO non‐TGF‐*β*1 mice versus the Smad3 WT non‐TGF‐*β*1 mice (^†^*P* < 0.05) (Fig. 3A). The presence of the *α*4 chain of collagen IV in the GBM is documented by immunofluorescence (Fig. 3B).

### Smad3‐deletion attenuates TGF‐*β*1‐mediated expansion of the mesangial matrix

**Figure 4.**
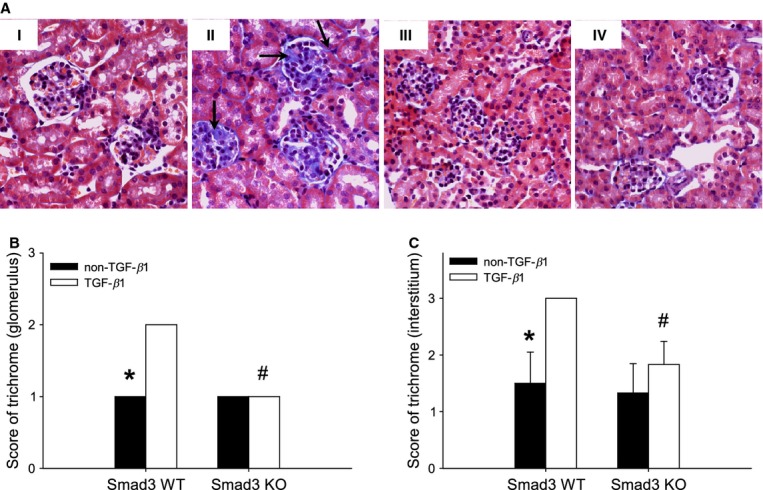
(A) Representative histological images of trichrome‐stained renal sections; (I) Smad3 WT non‐TGF‐*β*1, (II) Smad3 WT TGF‐*β*1, (III) Smad3 KO non‐TGF‐*β*1, and (IV) Smad3 KO TGF‐*β*1 mice. Note that glomerular as well as tubular interstitial accumulation of collagen is mainly seen in Smad3 WT TGF‐*β*1 mice (arrows) (original magnification: 40×). (B) A semiquantitative analysis of trichrome amount in the glomerulus is shown (*^#^*P* < 0.05 vs. Smad3 WT TGF‐*β*1 mice) (*n *= 6/group). (C) A semiquantitative analysis of trichrome amount in the interstitium is shown (*^#^*P* < 0.05 vs. Smad3 WT TGF‐*β*1 mice) (*n *= 6/group).

The volume fraction of the glomerular mesangium (*V*_*v*_(mes/glom)) is another marker of glomerulopathy. The *V*_*v*_(mes/glom) in non‐TGF‐*β*1 mice was independent of Smad3 genotype (*P *> 0.05) (Table 1). pTGF‐*β*1 overexpression increased the relative mesangial area in Smad3 WT mice (*P < 0.05) (Table 1), but not in Smad3 KO mice (Table 1). The observations were supported by the presence of collagen *α*1/*α*2(IV) deposits in the mesangium of Smad3 WT TGF‐*β*1 mice (Fig. 3C). In addition, finding of glomerular trichrome staining mainly in Smad3 WT TGF‐*β*1 mice (Fig. 4A and B) further supported the observation.

The total mesangial volume per glomerulus (*V*(mes, glom)) was reduced in Smad3 KO mice, although this was statistically significant only in Smad3 KO TGF‐*β*1 versus Smad3 WT TGF‐*β*1 mice (^†^*P* < 0.05) (Table 1). The smaller *V*_*n*_(glom) in Smad3 KO mice of both TGF‐*β*1 genotypes explains this finding.

### Smad3 KO mice are protected against TGF‐*β*1‐induced interstitial fibrosis and TBM thickening

**Figure 5.**
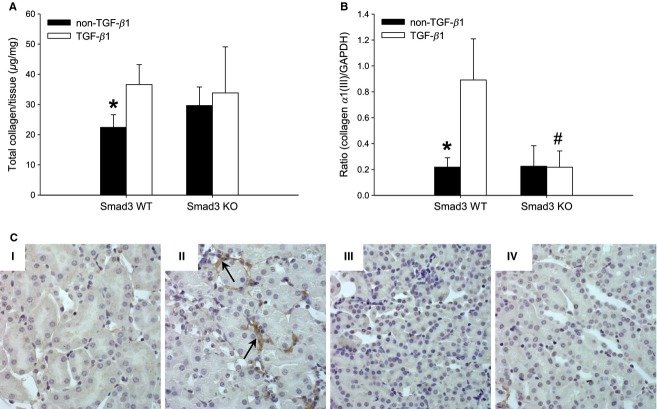
Total collagen amount, expression, and localization of collagen type III. (A) The renal total content of collagen estimated by the amount of hydroxyproline is upregulated in Smad3 WT TGF‐*β*1 mice versus Smad3 WT non‐TGF‐*β*1 mice (*n* = 13–16/group) (*P < 0.001). (B) The mRNA expression of collagen *α*1(III) is upregulated in Smad3 WT TGF‐*β*1 mice (*P < 0.001 vs. Smad3 WT TGF‐*β*1 mice). This TGF‐*β*1 effect is reversed by Smad3 KO (^#^*P *< 0.001 versus Smad3 WT TGF‐*β*1 mice) (*n* = 6–7/group). (C) Representative histology images of interstitial fibrillar collagen *α*1(III) (brown) in the tubulointerstitial space (original magnification: 40×). Note that localization of collagen *α*1(III) is best illustrated in Smad3 WT TGF‐*β*1 mice (arrows). Smad3 WT non‐TGF‐*β*1 (I), Smad3 WT TGF‐*β*1 (II), Smad3 KO non‐TGF‐*β*1 (III), and Smad3 KO TGF‐*β*1 (IV) mice.

Tubulointerstitial fibrosis is characterized by accumulation of ECM in the interstitial space, inflammatory changes, TBM thickening, and loss of renal tubules. To determine whether Smad3 deficiency affected TIF in vivo, we evaluated the collagen profile and the TBM thickness. pTGF‐*β*1 enhanced the total renal collagen content in Smad3 WT mice (*P < 0.001), but not in Smad3 KO mice (Fig. 5A). This observation was confirmed by histological evaluation demonstrating that mainly Smad3 WT TGF‐*β*1 mice exhibit trichrome staining in the tubulointerstitial space (Fig. 4AII and C). Part of the raised levels of total collagen in Smad3 WT TGF‐*β*1 was due to increased expression of interstitial fibrillar collagen *α*1(III) mRNA in Smad3 WT TGF‐*β*1 mice (*P < 0.001) (Fig. 5B). This was not found in Smad3 KO mice (Fig. 5B). As demonstrated by immunohistochemistry, collagen *α*1(III) deposits were localized in the tubulointerstitial space (Fig. 5C).

**Figure 6.**
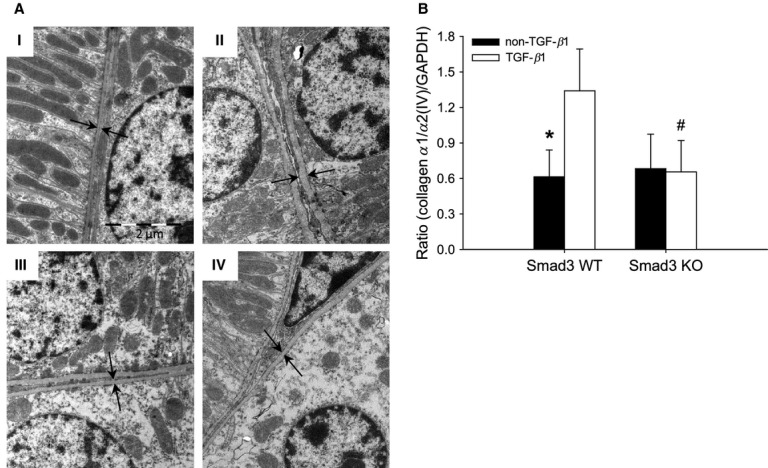
Evaluation of the tubular basement membrane (TBM). (A) TBM thickening determined by electron microscopy in each group of mice; Smad3 WT non‐TGF‐*β*1 (I), Smad3 WT TGF‐*β*1 (II), Smad3 KO non‐TGF‐*β*1 (III), and Smad3 KO TGF‐*β*1 (IV). Arrows indicate the TBM thickness. pTGF‐*β*1 overexpression caused TBM thickening in Smad3 WT mice (II). (B) Collagen *α*1/*α*2(IV) mRNA expression is increased in Smad3 WT TGF‐*β*1 mice (*P < 0.001), but not in Smad3 KO mice (*n* = 7–9/group). The mRNA expression of collagen *α*1/*α*2(IV) was lower in Smad3 KO TGF‐*β*1 mice versus Smad3 WT TGF‐*β*1 mice (^#^*P *< 0.05).

The TBM thickness was evaluated by electron microscopy and the expression of collagen *α*1/*α*2(IV), which is the most dominant collagen IV isoform of the TBM was evaluated. Overexpression of pTGF‐*β*1 caused a thickening of TBM in Smad3 WT mice (*P = 0.047), whereas this thickening was absent in Smad3 KO mice (Table 1 and Fig. 6A). In parallel, the presence of pTGF‐*β*1 enhanced collagen *α*1/*α*2(IV) mRNA expression in Smad3 WT mice (*P < 0.001), but not in Smad3 KO mice (Fig. 6B). Also, in line with the electron microscopy data, the amount of collagen *α*1/*α*2(IV) mRNA was lower in Smad3 KO TGF‐*β*1 mice versus Smad3 WT TGF‐*β*1 mice (^#^*P *< 0.05) (Fig. 6B).

### The expression of MMP‐2 is Smad3‐independent in vivo and gelatinase activity shows regional differences

**Figure 7.**
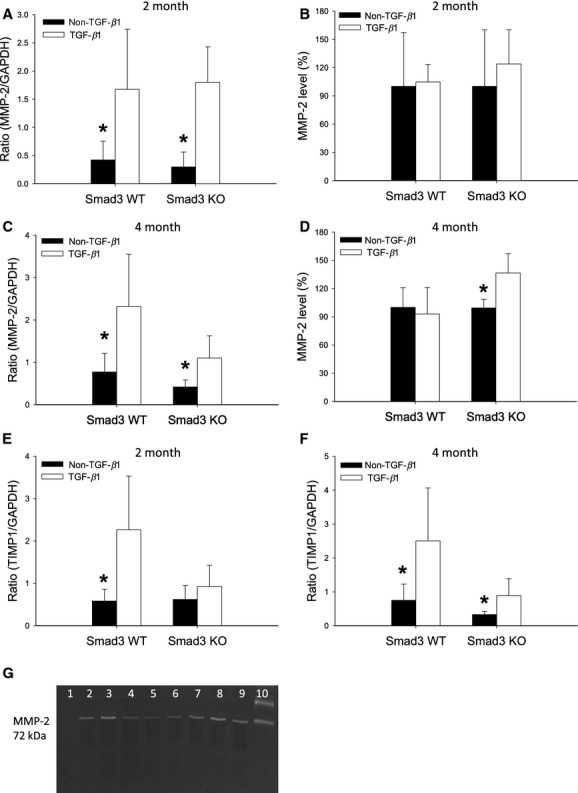
Renal expression of MMP‐2 and TIMP‐1. (A) MMP‐2 mRNA expression is independent of Smad3 in 2‐month‐old mice in vivo (*P = 0.001 vs. TGF‐*β*1 mice) (*n* = 12–13/group). (B) MMP‐2 level in 2‐month‐old mice (*n* = 3–5/group). (C) MMP‐2 mRNA expression is independent of Smad3 in 4‐month‐old mice in vivo (*P < 0.05 vs. TGF‐*β*1 mice) (*n* = 9–10/group). (D) Overexpression of TGF‐*β*1 increases the MMP‐2 level in 4‐month‐old Smad3 KO mice (*P = 0.02) (*n* = 5–7/group). The values from the Smad3 KO non‐TGF‐*β*1 mice are considered as 100%. (E) In 2‐month‐old animals TIMP‐1 mRNA expression is increased in Smad3 WT TGF‐*β*1 mice (*P < 0.05 vs. Smad3 WT TGF‐*β*1) (*n* = 10/group). (F) In 4‐month‐old mice the TIMP‐1 mRNA expression is elevated in both Smad3 WT TGF‐*β*1 and Smad3 KO TGF‐*β*1 (*P < 0.05) (*n* = 9–10/group). (G) Zymogram gel: lane 1: negative control; lane 2 and lane 6–9: Smad3 KO TGF‐*β*1 mice; lane 3–5: Smad3 KO non‐TGF‐*β*1 mice; lane 10: MMP‐2 (72 kDa) and ‐9 (92 kDa) standards. MMP‐2, Matrix metalloproteinase‐2; TIMP‐1, tissue inhibitors of metalloproteinase‐1.

**Figure 8.**
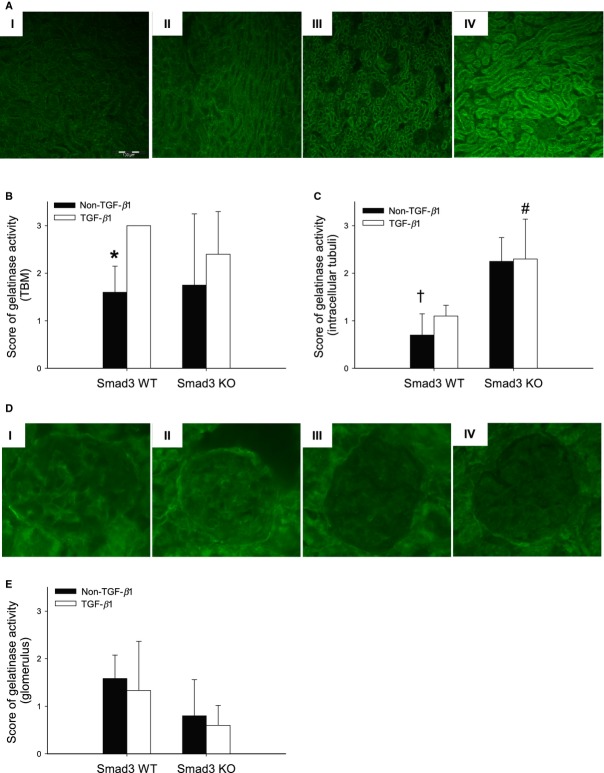
Localization of gelatinase activity in Smad3 WT (I, II), Smad3 KO (III, IV) non‐TGF‐*β*1 mice (I, III) and TGF‐*β*1 mice (II, IV). (A) The gelatinase activity (green) is highest intracellular of Smad3 KO TGF‐*β*1 mice versus the other three tested groups. (B and C) Score of gelatinase activity in the TBM and intracellular (in the tubular cells), respectively (*n* = 4–5/group) (*P = 0.008 vs. Smad3 WT TGF‐*β*1, ^#^*P* < 0.015 vs. Smad3 WT TGF‐*β*1 mice, and ^†^*P* = 0.002 vs. Smad3 KO non‐TGF‐*β*1 mice). (D) Localization of gelatinase activity (green) in the glomerulus. (E) A nonsignificant higher amount of activity are detected in the glomeruli of Smad3 WT versus Smad3 KO mice (*n* = 5–6/group). TBM, tubular basement membrane.

Our data indicate that TGF‐*β*1‐induced GBM and TBM thickening involve different molecular pathways. One explanation may be that mice show compartment‐specific regulation of signal molecules important for ECM turnover. Another possible explanation is cell‐specific functions of Smad2 and Smad3 in the regulation of genes implicated in ECM remodeling (Meng et al. 2010). To explore these possibilities further, two approaches were undertaken; first, the expression of MMP‐2 and MMP‐9, both of which have the ability to degrade basement membrane collagens (Lenz et al. 2000; Ronco et al. 2007), and their inhibitors TIMP‐1 and TIMP‐2 were analyzed. The presence of pTGF‐*β*1 induced an increase in MMP‐2 mRNA expression in both Smad3 WT and Smad3 KO animals at 2‐month of age (*P = 0.001) (Fig. 7A). The MMP‐2 level tended to parallel the augmented mRNA expression in Smad3 KO only (Fig. 7B). At the same age, TGF‐*β*1 led to an increase in TIMP‐1 mRNA in Smad3 WT mice (*P = 0.001) (Fig. 7E). TGF‐*β*1 had no effect on MMP‐9 and TIMP‐2 mRNA expression (data not shown). The analyses were repeated using tissue from 4‐month‐old mice. The same pattern of MMP‐2 mRNA expression was found (*P < 0.05) (Fig. 7C) and the MMP‐2 level paralleled the mRNA expression in Smad3 KO mice (*P < 0.05) (Fig. 7D and G). The TIMP‐1 mRNA expression was increased in both Smad3 WT TGF‐*β*1 and Smad3 KO TGF‐*β*1 mice versus non‐TGF‐*β*1 animals (*P < 0.05) (Fig. 7F). There was no effect on MMP‐9 and TIMP‐2 mRNA expression (data not shown). Second, the location of gelatinase activity was visualized by in situ zymography. Gelatinase activity (predominantly MMP‐2 and MMP‐9) was found in the TBM in all four groups of mice, and was increased in Smad3 WT TGF‐*β*1 animals versus Smad3 WT non‐TGF‐*β*1 mice (*P = 0.008) (Fig. 8A and B). In addition, strong intracellular gelatinase activity was seen in the epithelial lining of the tubules in both Smad3 KO groups (^#^*P *= 0.015, ^†^*P *= 0.002) (Fig. 8A and C). Finally, the gelatinase activity tended to be higher in the glomeruli of Smad3 WT versus Smad3 KO mice (Fig. 8D and E). However, this was not statistically significant. Thus, the gelatinase activity was clearly compartmentalized in Smad3 KO mice, being low in the glomeruli and high in tubular epithelial cells (Fig. 8A and C–E).

### Glomerular endothelial cells and mesangial cells differ in their response to TGF‐*β*1 in vitro

**Figure 9.**
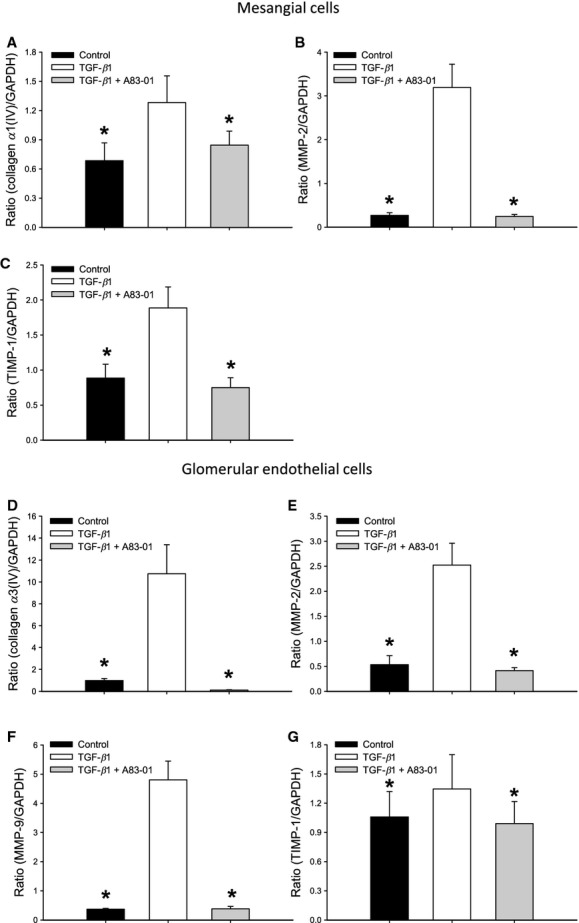
The response of murine mesangial (SV40 MES 13) and glomerular endothelial cells to TGF‐*β*1 (2 ng/mL) alone or in combination with the Smad2/3 inhibitor (A83‐01). TGF‐*β*1 stimulates collagen *α*1(IV) (A), MMP‐2 (B), and TIMP‐1 (C) mRNA expression in the mesangial cells and collagen type *α*3(IV) (D), MMP‐2 (E), MMP‐9 (F), and TIMP‐1 (G) mRNA expression in the glomerular endothelial cells. These effects are all reversed by addition of A83‐01 inhibitor (*P < 0.05 vs. TGF‐*β*1 group) (*n* = 6 wells/treatment/group). MMP‐2, matrix metalloproteinase‐2; MMP‐9, matrix metalloproteinase‐9; TIMP‐1, tissue inhibitors of metalloproteinase‐1.

We have shown that in Smad3 KO mice the gelatinase activity is low in the glomeruli and high in tubular epithelial cells. Furthermore, Smad3 KO prevents TGF‐*β*1‐induced TBM thickening and TGF‐*β*1‐induced mesangial matrix accumulation, but not GBM thickening. These findings suggest that the cellular components of the glomeruli may respond differently to knockdown of intracellular TGF‐*β*1 signal pathways versus cells in the extra glomerular parenchyma, and that the role of Smad3 in intracellular signaling differs in mesangial cells versus glomerular endothelial cells. Therefore, we tested the response of mesangial cells and endothelial cells to TGF‐*β*1 with and without the Smad2/3 inhibitor A83‐01 (Tojo et al. 2005). TGF‐*β*1 stimulated the mRNA expression of collagen *α*1(IV), MMP‐2, and TIMP‐1 in mesangial cells (all *P* < 0.05) (Fig. 9A–C). The effect of TGF‐*β*1 was fully reversed by A83‐01 in all cases (Fig. 9A–C). During the given conditions, TGF‐*β*1 had no effect on the mRNA expression of MMP‐9 and TIMP‐2 (data not shown).

In endothelial cells, TGF‐*β*1 stimulated the mRNA expression of collagen type *α*3(IV), MMP‐2, MMP‐9, and TIMP‐1 (*P < 0.05) (Fig. 9D–G). All expressions were neutralized by Smad2/3 inhibition (Fig. 9D–G). TGF‐*β*1 was without effect on the TIMP‐2 mRNA expression (data not shown). Thus, mesangial cells and glomerular endothelial cells do not respond in the same way to TGF‐*β*1 exposure regarding MMP‐9 expression.

To identify whether these TGF‐*β*1‐sensitive effector molecules were regulated through Smad2 or Smad3 the mesangial and endothelial cells were transfected with siRNA. Based on the results obtained from two cell lines, siSmad3‐2 (Smad3‐murine‐4492) was selected for the experiments (data not shown).

**Figure 10.**
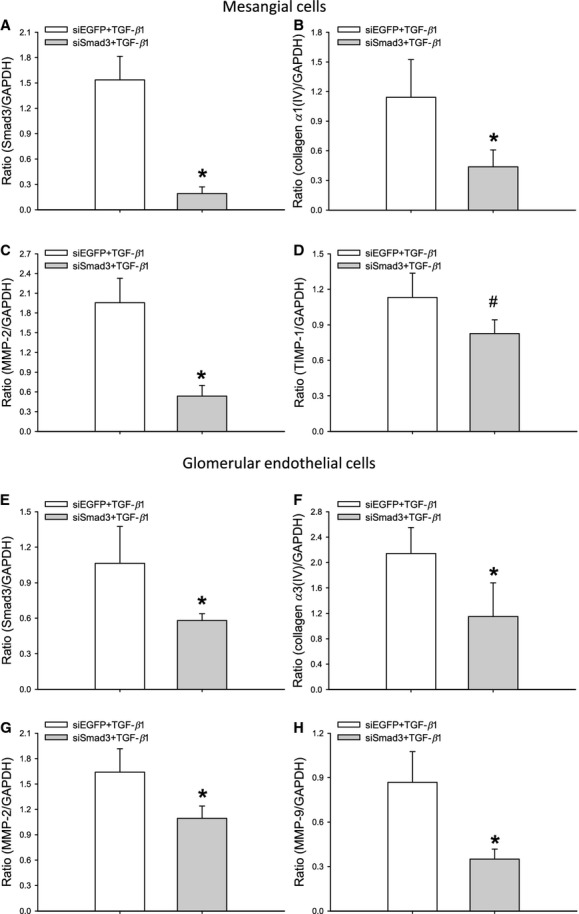
Knockdown of Smad3 with siRNA (siSmad3‐2) in murine mesangial and glomerular endothelial cells. In the mesangial cells, real‐time PCR analysis shows that knockdown of Smad3 attenuates TGF‐*β*1‐induced expression of collagen *α*1(IV) (B) and MMP‐2 (C) mRNA versus the control (siEGFP) (*P < 0.05), whereas TIMP‐1 (D) is partly blocked (^#^*P *= 0.07) (*n* = 11–12 wells/treatment/group). In the glomerular endothelial cells, knockdown of Smad3 blocks TGF‐*β*1‐induced collagen *α*3(IV) (F), MMP‐2 (G), and MMP‐9 (H) mRNA expression (*P ≤ 0.05) (*n* = 7 wells/treatment/group). MMP‐2, matrix metalloproteinase‐2; MMP‐9, matrix metalloproteinase‐9; TIMP‐1, tissue inhibitors of metalloproteinase‐1.

Smad3 knockdown in mesangial cells attenuated TGF‐*β*1‐induced collagen *α*1(IV) and MMP‐2 mRNA expression (*P < 0.05), which therefore is Smad3‐dependent (Fig. 10B and C). However, the reduction in TIMP‐1 mRNA expression was not statistically significant (^#^*P *= 0.07) (Fig. 10D). Control experiments using fibronectin mRNA expression as endpoint showed that TGF‐*β*1‐induced fibronectin release in mesangial cells transfected with siEGFP (*n* = 6 wells/treatment/group) was similar to cells exposed to TGF‐*β*1 alone (*n* = 6 wells/treatment/group) (*P* > 0.05) (data not shown). Furthermore, no effect of siEGFP alone on the expression of fibronectin was observed (*n* = 6 wells/treatment/group) (*P* > 0.05) (data not shown).

In endothelial cells, we found that Smad3 knockdown attenuated TGF‐*β*1‐induced collagen *α*3(IV), MMP‐2, and MMP‐9 expression (*P < 0.05) (Fig. 10F–H), which therefore is Smad3‐dependent, whereas TIMP‐1 mRNA expression was unaffected and thereby mediated through Smad2 (data not shown). Control experiments using fibronectin mRNA expression as endpoint demonstrated that TGF‐*β*1‐induced fibronectin release in endothelial cells transfected with siEGFP (*n* = 7 wells/treatment/group) was similar to cells exposed to TGF‐*β*1 and Lipofectamine alone (*n* = 5 wells/treatment/group) (*P* > 0.05) (data not shown). In summary, neither the transfection agent nor the siEGFP influence the cellular responses to TGF‐*β*1.

## Discussion

The aim of this study was to investigate the consequence of Smad3 deficiency in TGF‐*β*1‐induced chronic kidney disease with special emphasis on ECM turnover and MMP regulation. We report the following major observations: (I) Smad3 KO mice exhibit low BW, albuminuria, reduced megalin mRNA expression, and spatial distribution of renal gelatinase activity, being low in glomeruli and high intracellular activity in the tubules. (II) Smad3 deficiency prevents TGF‐*β*1‐induced TBM thickening, TIF, and mesangial matrix expansion, but not GBM thickening. (III) Smad3 deficiency attenuates TGF‐*β*1‐induced mRNA expression of collagen *α*1/*α*2(IV) in mesangial cells and whole kidney tissue. (IV) The transcriptional regulatory signaling of TGF‐*β*1 on expression of particular genes differs in different renal compartments; (a) the regulation of collagen *α*3(IV) gene expression is Smad3‐dependent in glomerular endothelial cells, but Smad3‐independent when analyzing whole kidney tissue. (b) The regulation of MMP‐2 gene expression is Smad3‐dependent in mesangial cells and endothelial cells, but Smad3‐independent when analyzing whole kidney tissue, and finally, (c) the regulation of TIMP‐1 gene expression is Smad3‐independent in glomerular endothelial cells, but partly Smad3‐dependent when analyzing mesangial cells and whole kidney tissue.

The original Smad3‐deficient mice were generated by disruption of the first exon, including the ATG sequence, against a background of 129/C57BL/6 hybrid mice (Datto et al. 1999). They were backcrossed with TGF‐*β*1 transgenic animals (BALB/cA) (Wogensen et al. 1999). The produced Smad3 KO animals have a reduced growth rate, and a subpopulation exhibits torqued paws, kyphosis, and rib cage malformations, as previously described (Datto et al. 1999). A lower BW is also reported for other Smad3‐deficient mice made by disruption of exon 2 (Zhu et al. 1998) or exon 8 (Fujimoto et al. 2003; Inazaki et al. 2004; Yang et al. 1999). In addition to reduced weight, the Smad3 KO animals have smaller glomerular volume, less total glomerular mesangium, and thinner GBM than their Smad3 WT siblings do. This is in contrast to Wang et al. who report that Smad3 WT and KO mice have similar weights, *V*_*n*_(glom) and *V*_*v*_(mes/glom) (Wang et al. 2007). Importantly, the KW/BW and the *V*_*v*_(mes/glom) ratios are similar, indicating that the glomerular dimensions follow the size of the animal and therefore not specifically hampered by Smad3 deficiency. In addition, the thinner GBM is most likely explained by the reduced size of the animals, but we recognize that specific effects of Smad3 deficiency are possible.

The Smad3 KO mice spontaneously develop albuminuria, indicating that Smad3 may influence renal protein handling. Megalin is an endocytic receptor that is involved in tubular albumin reabsorption. The Smad3 KO mice exhibit decreased megalin mRNA expression, suggesting that Smad3 may influence transcriptional control of the megalin gene or mRNA stability. An influence of Smad3 KO on tubular function is not surprising as the intracellular TGF‐*β*1 signaling cascade is present in the tubules. Our in vivo observation is in contrast to the finding that TGF‐*β*1‐mediated Smad2 and Smad3 phosphorylation reduces megalin‐cubilin‐receptor‐mediated endocytosis of albumin by proximal tubule cells in vitro (Gekle et al. 2003). However, intracellular activation of MMP is reported and may be an indication of intracellular protein turnover (Hadler‐Olsen et al. 2011). Thus, in Smad3 KO mice the translocation of megalin to the epithelial surface may be affected by the presence of increased levels of intracellular gelatinase activity. The podocytes contribute to the filtration barrier in the glomeruli, not only by production of ECM but also by virtue of the properties of their foot processes and slit diaphragm (Reeves et al. 1978; Welsh and Saleem 2012). Changes herein may also contribute to the observed albuminuria in the Smad3 KO mice. Although the foot processes seem normal and that the podocytes display Smad3/Smad2 gene redundancy and therefore may be unaffected by Smad3 deficiency (Wu et al. 2005), it is acknowledged that functional changes in the podocytes cannot be excluded.

In Smad3 KO animals, the TGF‐*β*1‐induced reduction in BW, the increase in *V*_*v*_(mes/glom), total renal collagen content, and the total renal mRNA expression of collagen types *α*1(III) and *α*1/*α*2(IV) are attenuated. In contrast, the TGF‐*β*1‐induction of total renal MMP‐2 mRNA expression is clearly Smad3‐independent. This is as expected and confirms the value of our model in the study of TGF‐*β*1 signaling in vivo (Datto et al. 1999; Fujimoto et al. 2003; Sato et al. 2003; Wang et al. 2007; Yang et al. 1999).

A significant finding is that Smad3 deficiency prevents TGF‐*β*1‐induced mesangial expansion, TBM thickening, and TIF. The reduction in TIF is partly explained by attenuation of the renal mRNA expression of interstitial fibrillar collagen type *α*1(III), whereas blockage of TGF‐*β*1‐mediated transcriptional regulation of collagen type *α*1/*α*2(IV) contributes to the prevention of TGF‐*β*1‐induced mesangial expansion and TBM thickening. This is supported by our finding that Smad3 knockdown of mesangial cells in vitro inhibits TGF‐*β*1‐induced collagen type *α*1(IV) mRNA expression and that Smad3 deficiency reduces TGF‐*β*1‐induced collagen type *α*1/*α*2(IV) expression in the kidney in vivo.

The mechanisms leading to TGF‐*β*1‐induced GBM thickening is Smad3‐independent. The major collagen type IV in the GBM is *α*3/*α*4/*α*5(IV) (Miner 1999; Zeisberg et al. 2002). The direct exposure of endothelial cells to TGF‐*β*1 leads to a Smad3‐dependent stimulation of collagen *α*3(IV), MMP‐2, and MMP‐9 mRNA expression. Therefore, one should assume that Smad3 KO should reverse also TGF‐*β*1‐induced GBM thickening. However, it should be considered that the endothelial TIMP‐1 expression is Smad3‐independent in vitro and that the glomerular gelatinase activity is low in Smad3 KO animals in vivo. Furthermore, it is well‐known that the podocytes also contribute to the GBM and exhibit Smad2/3 redundancy (Miner 2012). Therefore, the biochemical consequences of Smad3 deficiency on GBM composition are most likely dependent on an interaction between the endothelial cells, the podocytes, and the microenvironment. Thus, in a microenvironment of low gelatinase activity combined with elevated RNA expression of TIMP‐1 and continuous production of GBM components by the podocytes, the GBM stays widened in Smad3 KO TGF‐*β*1 mice.

Disruption of Smad3 prevented TGF‐*β*1‐induced mesangial matrix expansion in a situation with low glomerular gelatinase activity. This observation is in contrast to the continued GBM thickening in Smad3‐deficient mice. However, the reduced accumulation of mesangial ECM is partly explained by the Smad3‐dependent expression of collagen *α*1(IV) in mesangial cells in vitro. Thus, as opposed to the components of the GBM, the production of mesangial matrix proteins is reduced in Smad3 KO mice compared to the WT mice. The fact that mesangial and glomerular endothelial cells are derived from different cell precursors, may explain the diverse response to TGF‐*β*1.

The impact of MMPs on ECM turnover can be either positive or negative to disease progression (Tan and Liu 2012). However, the outcome is also dependent on biological effects of MMPs not related to ECM metabolism and the balance between MMPs and their inhibitors. A majority of studies in patients with kidney diseases as well as animal models hereof demonstrate that the renal content of MMP‐2 and MMP‐9 are increased (Gharagozlian et al. 2009; Krag et al. 2007; Lu et al. 2011; Tan and Liu 2012; Thrailkill et al. 2009; Wang et al. 2010; Williams et al. 2011). Whether this is a causal relation, an epiphenomenon or a compensatory mechanism is still under investigation. Interestingly, recent studies demonstrate that inhibition of MMPs can reduce renal fibrosis (Wang et al. 2010; Williams et al. 2011). The expression of MMP‐2 mRNA is increased without a parallel rise in the MMP‐2 level in 2‐month‐old Smad3 WT TGF‐*β*1 mice and in 4‐month‐old WT TGF‐*β*1 animals. This may partly be due to MMP‐2 inhibition by TIMP‐1. It should be considered, however, that only TIMP‐1 mRNA expression was measured and that other factors may contribute as well. The expression of MMP‐2 mRNA is also increased in Smad3 KO TGF‐*β*1 mice. In 4‐month‐old mice, this is paralleled by a rise in the MMP‐2 level. Although TIMP‐1 mRNA is increased in Smad3 KO TGF‐*β*1 mice, this may be insufficient to counteract MMP‐2 activities and consequently, the balance is shifted toward MMP‐2 dominance, which may influence the renal phenotype in Smad3 KO TGF‐*β*1 mice.

While we found that the overall MMP‐2 mRNA expression and level were Smad3‐independent when analyzing whole kidney tissue, in situ zymography elucidated that the spatial localization of the gelatinase activity differed between Smad3 WT mice and Smad3 KO animals. The main observation was a high level of tubular gelatinase activity in Smad3 KO mice. Preliminary experiments employing rat tubular cells show that MMP‐2 knockdown in vitro increases the expression of collagen *α*1/*α*2(IV) (Pedersen et al. unpublished observations). This suggests a link between intracellular MMP‐2 expression and regulation of collagen *α*1/*α*2(IV) gene transcription. One may postulate that the observed Smad3‐independent MMP‐2 expression and level observed in whole kidney tissue might be in contrast to the data obtained by in situ zymography. However, in situ zymography localizes all activated enzymes with gelatinase activity and possibly not only MMP‐2 and MMP‐9. Additionally, the reduced gelatinase activity in the glomeruli of Smad3 KO can easily be outweighed by the high intracellular protease activity in the tubular epithelial cells.

In summary, we show that deletion of Smad3 protects the kidney from developing TGF‐*β*1‐induced TIF, mesangial matrix expansion, and TBM thickening, but not GBM thickening. In addition, Smad3 KO hampers renal protein handling, which is associated with decreased megalin mRNA expression and intracellular high MMP activity. In general, the favorable effects of Smad3 deficiency can be explained by reduced deposition of collagen subtypes. The cell‐specific changes of MMP expression can be a result of altered TGF‐*β*1 signaling. The poor effect on the GBM may be due to the presence of Smad3‐independent TGF‐*β*1 signaling in the endothelial cells as found for TIMP‐1 and the contribution of the podocytes to the GBM. In conclusion, an important lesson of this study is that the role of Smad3 in ECM modeling and MMP regulation is cell‐specific. This may be a shortcoming of the postulated beneficial therapy targeting Smad3 in patients with chronic kidney diseases, and call for cell‐targeted intervention strategies.

## Acknowledgments

We are indebted to Lotte Arentoft, Ulla Hovgaard, Lone Lysgaard, Anette Larsen, and Annette Berg for their technical assistance and to the staff at the animal facility, Faculty of Health Sciences, University of Aarhus, Denmark. Thanks to Nese Akis and Hans‐Joachim Anders for the gift of the glomerular endothelial cell line.

## Conflict of Interest

None declared.
